# Mesoporous cerium oxide nanospheres for the visible-light driven photocatalytic degradation of dyes

**DOI:** 10.3762/bjnano.5.60

**Published:** 2014-04-24

**Authors:** Subas K Muduli, Songling Wang, Shi Chen, Chin Fan Ng, Cheng Hon Alfred Huan, Tze Chien Sum, Han Sen Soo

**Affiliations:** 1 Division of Chemistry and Biological Chemistry, School of Physical and Mathematical Sciences, Nanyang Technological University, Singapore 637371; 2 Department of Chemistry, National University of Singapore, 10 Kent Ridge, Singapore 119260; 3 Division of Physics and Applied Physics, School of Physical and Mathematical Sciences, Nanyang Technological University, Singapore 637371; 4 Institute of High Performance Computing, Agency for Science, Technology and Research, 1 Fusionopolis Way, #16-16 Connexis, Singapore 138632; 5 Energy Research Institute @ NTU (ERI@N), 1 CleanTech Loop, Singapore 637141; 6 Singapore-Berkeley Research Initiative for Sustainable Energy (SinBeRISE), 1 Create Way, Singapore 138602

**Keywords:** cerium oxide, dye degradation, mesoporous, photocatalysis, visible light

## Abstract

A facile, solvothermal synthesis of mesoporous cerium oxide nanospheres is reported for the purpose of the photocatalytic degradation of organic dyes and future applications in sustainable energy research. The earth-abundant, relatively affordable, mixed valence cerium oxide sample, which consists of predominantly Ce_7_O_12_, has been characterized by powder X-ray diffraction, X-ray photoelectron and UV–vis spectroscopy, and transmission electron microscopy. Together with N_2_ sorption experiments, the data confirms that the new cerium oxide material is mesoporous and absorbs visible light. The photocatalytic degradation of rhodamin B is investigated with a series of radical scavengers, suggesting that the mechanism of photocatalytic activity under visible-light irradiation involves predominantly hydroxyl radicals as the active species.

## Introduction

The degradation of organic pollutants by affordable and effective chemical methods is an acute problem that has been tackled by advanced oxidation processes [1]. The photocatalytic production of reactive oxygen species by using semiconductor technology has emerged as a sustainable and promising route for such advanced oxidation processes [2–6]. In these photocatalytic processes, based on TiO_2_ for example, radiation larger than the band gap is absorbed to promote an electron from the valence to the conduction band [2–4]. The resultant strongly oxidizing, valence band holes (*h*^+^) and reducing, conduction band electrons (*e*^–^) are short-lived under ambient conditions and react with water and air to form reactive oxygen species such as ^•^OH, ^•^OOH, H_2_O_2_, and O_2_^−^ for example [1–2,4–6]. These reactive oxygen species can subsequently decompose organic pollutants. Recent developments in nanotechnology have enhanced the performance of photocatalytic and solar energy absorption processes by providing higher surface areas and more effective charge separation in semiconductor materials on the nanoscale. In fact, the commercially available Degussa P25 mixed-phase TiO_2_ is commonly employed as a benchmark in photocatalysis for applications ranging from dye-sensitized solar cells to the oxidative degradation of pollutants [7–11]. Despite being cheap, chemically robust, and generally non-toxic, TiO_2_ has a wide band gap of more than 3.0 eV, which means that photocatalytic processes that use TiO_2_ as the sensitizer can only absorb UV radiation (≈5% of the solar spectrum) [2–4,7–8]. Moreover, the valence band of TiO_2_ is strongly oxidizing whereas the conduction band level is only mildly reducing, which results in a low energy-conversion efficiency since most of the oxidation potential is wasted thermally. A number of other metal oxide semiconductors have been explored for the visible-light driven photocatalytic degradation of pollutants and microbes, such as bismuth oxides [5–6] and cerium oxides [12–13]. CeO_2_ specifically has been applied in a number of sustainable energy applications lately, including oxidative catalysis, hydrogen storage, and solar thermal dissociation of water and CO_2_ [14–18].

Cerium oxides with oxygen vacancies represent an underexplored area of nanotechnology with the potential to provide visible-light absorbing photocatalysts [13,19–21]. Cerium is relatively earth-abundant and the oxides, including Ce_2_O_3_ and Ce_7_O_12_, are known to have band gaps in the visible region [13,19–21]. Our team has maintained a keen interest in alternative affordable, earth-abundant, visible light absorbing metal oxides to be used in two-photon ‘Z-schemes’ for dye-sensitized photoelectrosynthesis cells (DSPECs) [22–24]. To be employed in DSPECs, high surface areas for dye adsorption and an efficient charge conduction are critical properties [22–24]. As part of the preliminary investigations into this field, we communicate herein the preparation of high surface area, mesoporous cerium oxide nanospheres, which is a mixed phase of Ce_7_O_12_ and CeO_2_, and can absorb visible light to photocatalytically degrade dyes such as rhodamine B (RhB). The materials characterization of the cerium oxide nanospheres and some mechanistic insights into the photocatalytic process are presented.

## Findings

**Figure 1:**
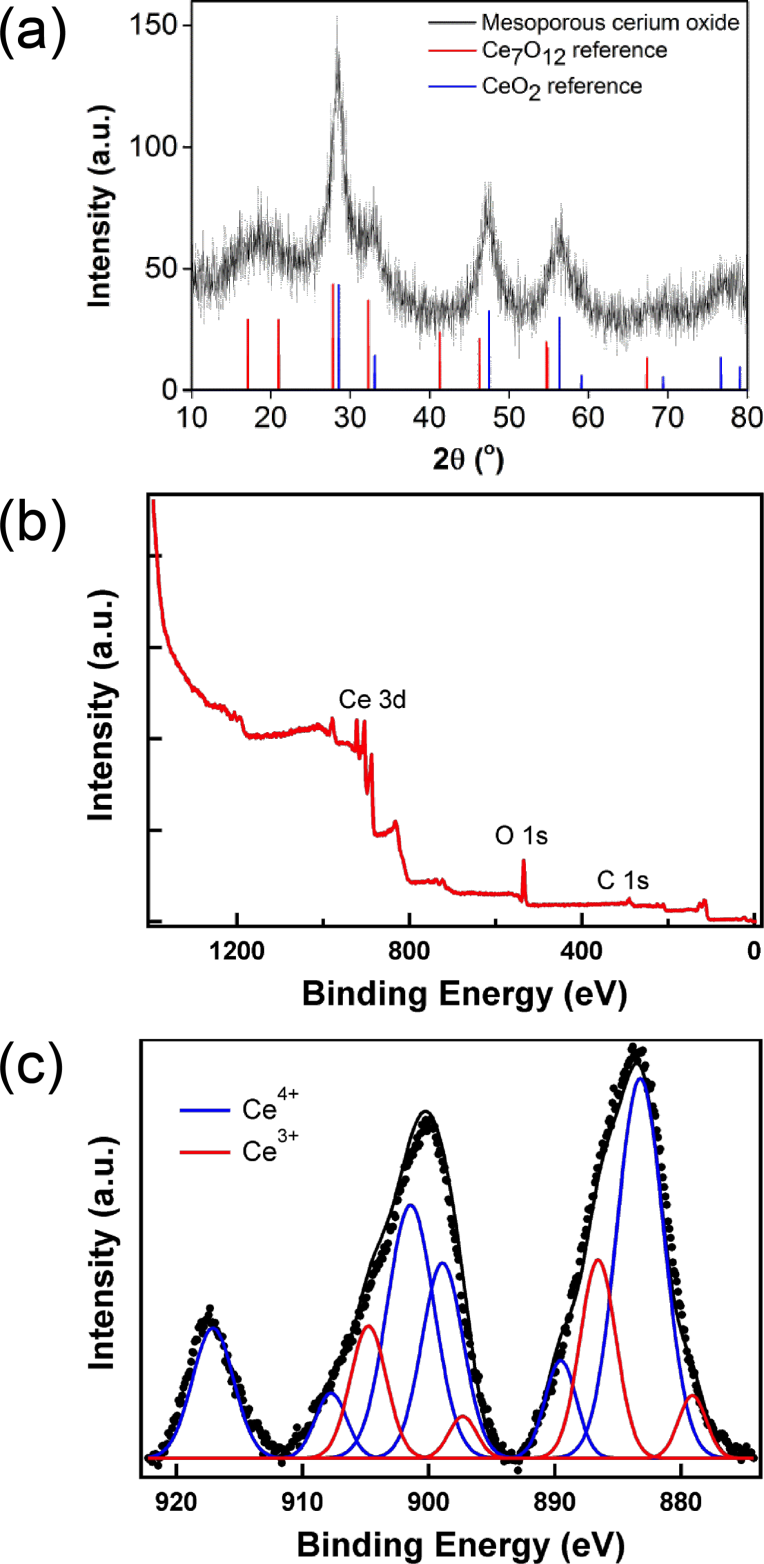
(a) Powder XRD pattern of cerium oxide nanospheres. (b) Wide-scan XPS survey spectrum. (c) High-resolution XPS spectrum of mesoporous cerium oxide (black circles) with the overall fit (black) and the fits to Ce^4+^ (blue) and Ce^3+^ (red).

Polycrystalline Ce_7_O_12_ samples have been previously synthesized, but harsh conditions (up to 1030 °C) by reduction of CeO_2_ with CO were employed [25–26]. Instead, mild, surfactant-free solvothermal conditions were used to prepare mesoporous cerium oxide with oxygen vacancies. A solution of ceric ammonium nitrate (CAN) in ethylene glycol and isopropanol as the solvent and reductant was heated up to 130 °C to yield mesoporous cerium oxide nanospheres after work-up. The powder X-ray diffraction (XRD) pattern (Figure 1a) indicates that the as-prepared cerium oxide material can be indexed to a superposition of hexagonal Ce_7_O_12_ (JCPDS File No. 71-0567) and cubic CeO_2_ (JCPDS File No. 81-0792) phases [14,26–27]. The peaks cannot be attributed to Ce(OH)_3_ or Ce_2_O_3_ phases [13,28], and confirm that the material contains a mixed phase. The considerable broadening of the peaks suggest that the domain sizes of the nanocrystalites are small, and has been estimated to be 4.8 nm ((211) plane, 2θ = 28.3°) by the Scherrer equation [29].

In order to confirm the valence states of Ce and quantify their relative ratios in the prepared cerium oxide, X-ray photoelectron spectroscopy (XPS) experiments with monochromatic Al Kα radiation (*h*ν = 1486.7 eV) were conducted. Unlike CeO_2_, in which the Ce atoms are all in the oxidation state 4+, the Ce atoms in Ce_7_O_12_ consist of both Ce^3+^ and Ce^4+^ valence states. The wide-scan survey spectrum in Figure 1b only shows Ce 3d, O 1s, and C 1s signals, and no other signals. The presence of the C 1s signal is probably from residual organic solvents or from air. This C 1s signal was used to calibrate the binding energy of the Ce 3d peaks. The high-resolution spectrum of the Ce 3d core states is illustrated in Figure 1c. Neither Ce^4+^ nor Ce^3+^ alone could give a satisfactory fitting to the spectrum in Figure 1c. Instead, the fitting of the Ce 3d spectrum required five components derived from both Ce^3+^ and Ce^4+^. There are two components (red) from Ce^3+^. The principal peak is at 886.4 eV and a 4f^0^ to 4f^1^v (v denotes valence hole) shake-down peak is at 879.9 eV [30]. The Ce^4+^ component consists of three peaks (blue). The peaks at 889.3 eV and 883.0 eV are the principal and 4f^1^v to 4f^2^v^2^ shake-down peaks from the 4f^1^v electronic configuration. The highest binding energy peak at 898.7 eV is from the 4f^0^ electronic configuration [30]. The binding energy of these peaks is in good agreement with those found in the literature [19–20,31]. However, by integrating the area under the fitted peaks, the concentration of Ce^3+^ is only 23%, which deviates from the predicted stoichiometric value (57%). This observation suggests that the Ce_7_O_12_ phase is mixed with some CeO_2_ phase on the surface. The nominal molecular formula of the material based on the XPS data is CeO_1.89,_ comprising of around 54% Ce_7_O_12_ and 46% CeO_2_. The mixture of two crystalline forms is also observed in our XRD measurements and TEM results (vide infra).

**Figure 2:**
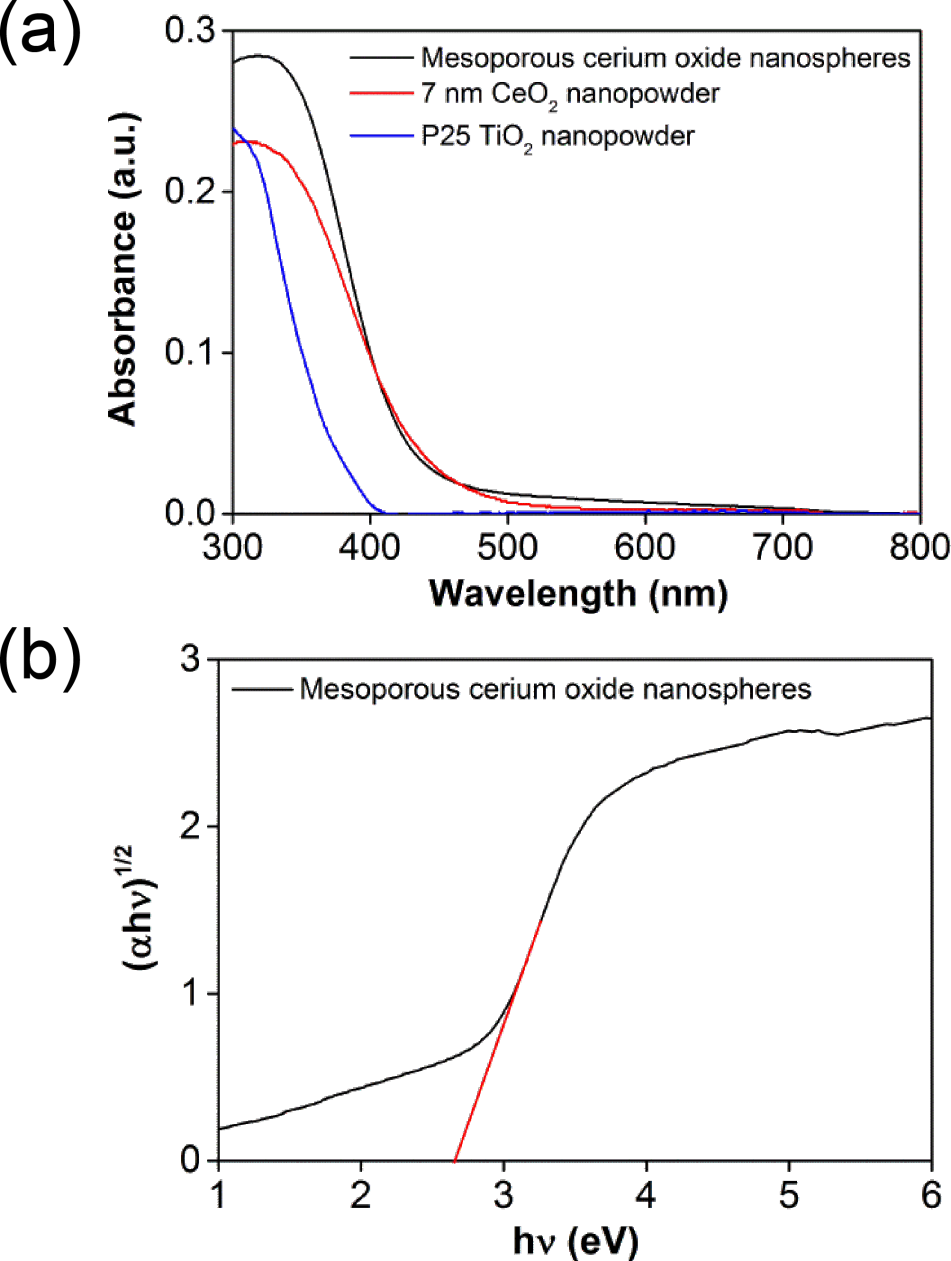
(a) UV–vis diffuse reflectance spectra of cerium oxide nanospheres (black), 7 nm CeO_2_ (red) nanopowder, and P25 TiO_2_ (blue). (b) Tauc plot for cerium oxide to obtain the band gap.

The UV–vis diffuse reflectance spectra (subjected to a Kubelka–Munck transformation) of the cerium oxide nanospheres, CeO_2_ (commercially available 7 nm) nanopowder, and TiO_2_ (commercially available Degussa P25) nanoparticles are illustrated in Figure 2a. As expected, the cerium oxide sample displayed stronger visible light absorption than both commercially available 7 nm CeO_2_ and P25 TiO_2_ nanomaterials. The estimated band gap from the Tauc plot is approximately 2.7 eV (Figure 2b), which corresponds to an absorption edge in the blue region (460 nm). The reduced band gap compared to CeO_2_ can be attributed to the presence of oxygen vacancies, as previously reported [32]. The enhanced visible light absorption has been exploited for driving the photocatalytic degradation of RhB in aqueous solutions (vide infra).

**Figure 3:**
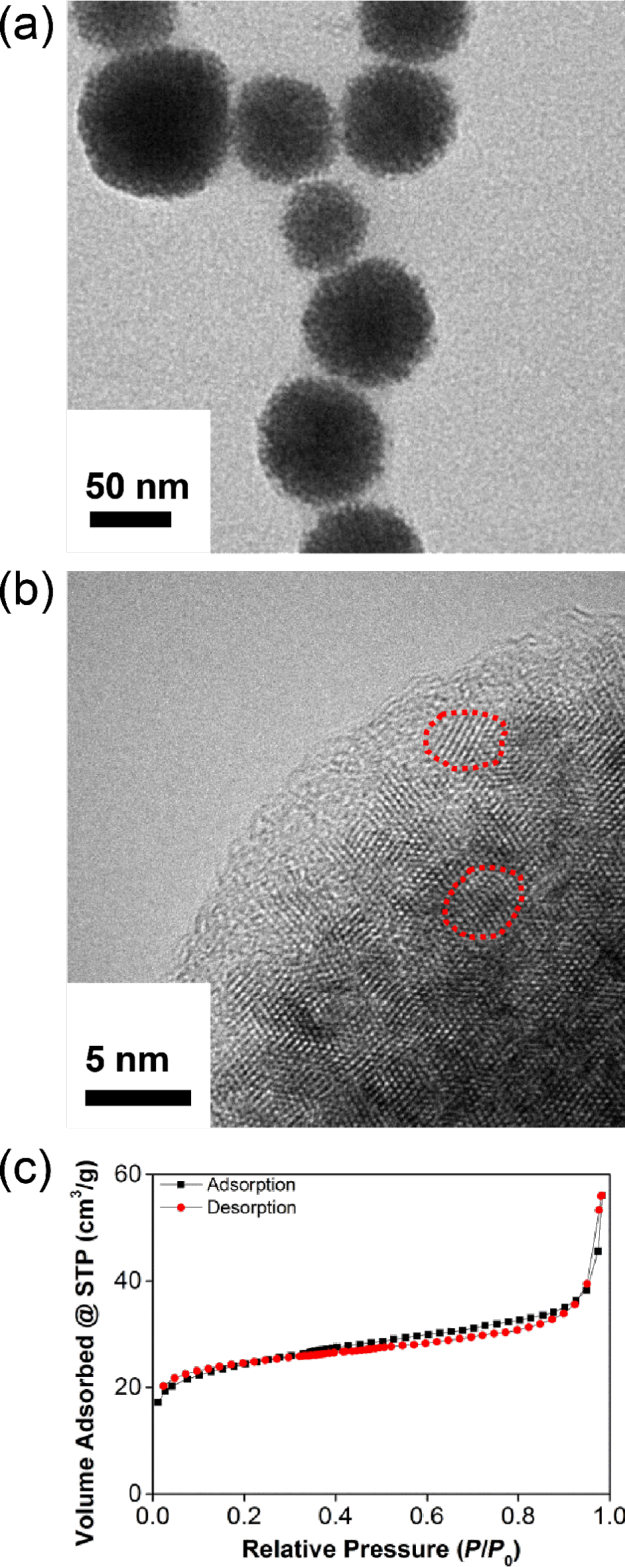
(a) TEM and (b) HRTEM images of the mesoporous cerium oxide nanospheres. (c) Nitrogen adsorption–desorption isotherm of the mesoporous cerium oxide nanospheres.

The transmission electron microscopy (TEM) images of the cerium oxide sample supported the mesoporous nature and nanosphere morphology of the material (Figure 3a). The material has fairly monodisperse nanospheres with diameters of 50–70 nm. Each nanosphere consists of an irregular mesoporous structure that is an aggregate of small nanocrystalline domains. The high-resolution TEM images confirm that the cerium oxide consists of crystalline domains, 4–5 nm in size (red dotted ring), that can be indexed to Ce_7_O_12_ and CeO_2_ (Figure 3b). Nitrogen sorption experiments were conducted to ascertain the average surface area and pore size distribution of the material. The nitrogen adsorption–desorption isotherm of the cerium oxide sample (Figure 3c) shows a type-II curve and the surface area of the sample is 93 m^2^ g^−1^ as calculated by the Brunauer–Emmett–Teller (BET) method. The average pore size determined by a Barrett–Joyner–Halenda (BJH) analysis is 3 nm, confirming the mesoporous nature of the cerium oxide sample.

**Figure 4:**
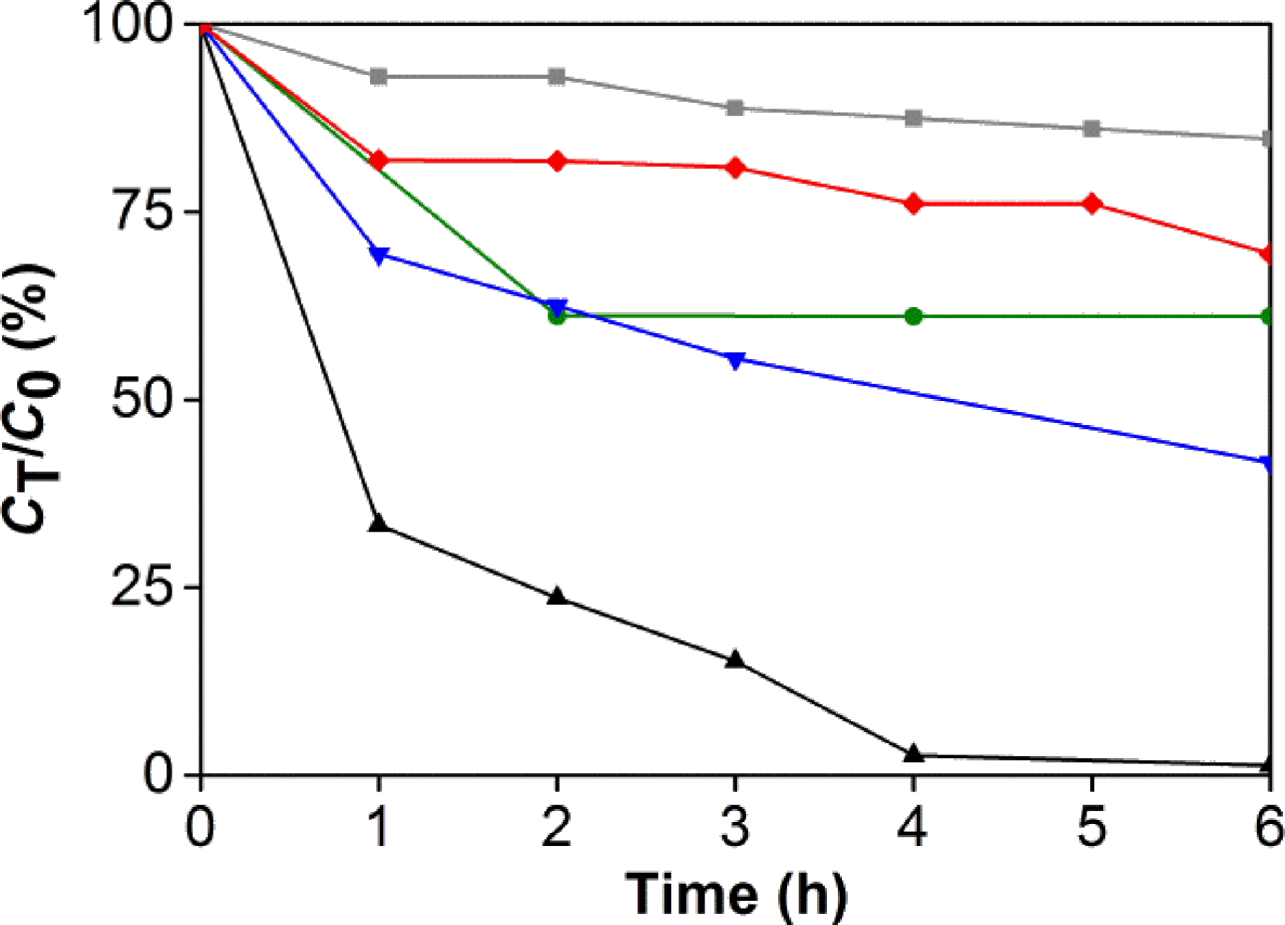
Comparison of RhB concentrations over time at 554 nm, after photocatalytic degradation with mesoporous cerium oxide under light (black) and in the dark with no equilibration (green), 7 nm CeO_2_ (red), P25 TiO_2_ (blue), and with no catalyst (grey).

The photocatalytic behavior at visible-light irradiation of the cerium oxide sample has been probed by the photodegradation of the suspected carcinogenic dye rhodamine B (RhB). A colloidal mixture of cerium oxide and RhB has been stirred and irradiated with AM 1.5 solar intensity light after equilibration in the dark for 30 min. A standard glass filter has been applied to transmit only wavelengths larger than 420 nm, to demonstrate photocatalytic properties under ambient conditions. The UV–vis spectral changes of the colloidal mixture illustrated in Figure 4 clearly shows the degradation of RhB over time, with the dye being completely decomposed within 6 h. In comparison, RhB is only decomposed to 50% or less after irradiation under the same conditions with the commercially available P25 TiO_2_ and 7 nm CeO_2_ nanopowder. The visible-light photocatalytic degradation of organic compounds with wide band-gap materials, by a ligand-to-metal charge-transfer mechanism after adsorption [33], has been reported and may be a contributing factor for the activity of TiO_2_ and CeO_2_. In the absence of light, some of the RhB adsorbs on the cerium oxide (green). The mesoporous cerium oxide sample is patently a more effective agent for the photocatalytic degradation of RhB under visible light and ambient conditions after equilibration, and the activity cannot be accounted to the presence of CeO_2_ or adsorption alone. Gas chromatography mass spectrometry (GC–MS) and electrospray ionisation mass spectrometry (ESI–MS) were used to identify some of the organic products during the course of the 6 h irradiation (see Supporting Information File 1). These included *N*-hydroxylated desethyl rhodamine B, phthalic acid, and even ring-opened products [6]. The composition of the degradation products alludes to oxidative decomposition by reactive oxygen species, such as hydroxyl radicals.

**Figure 5:**
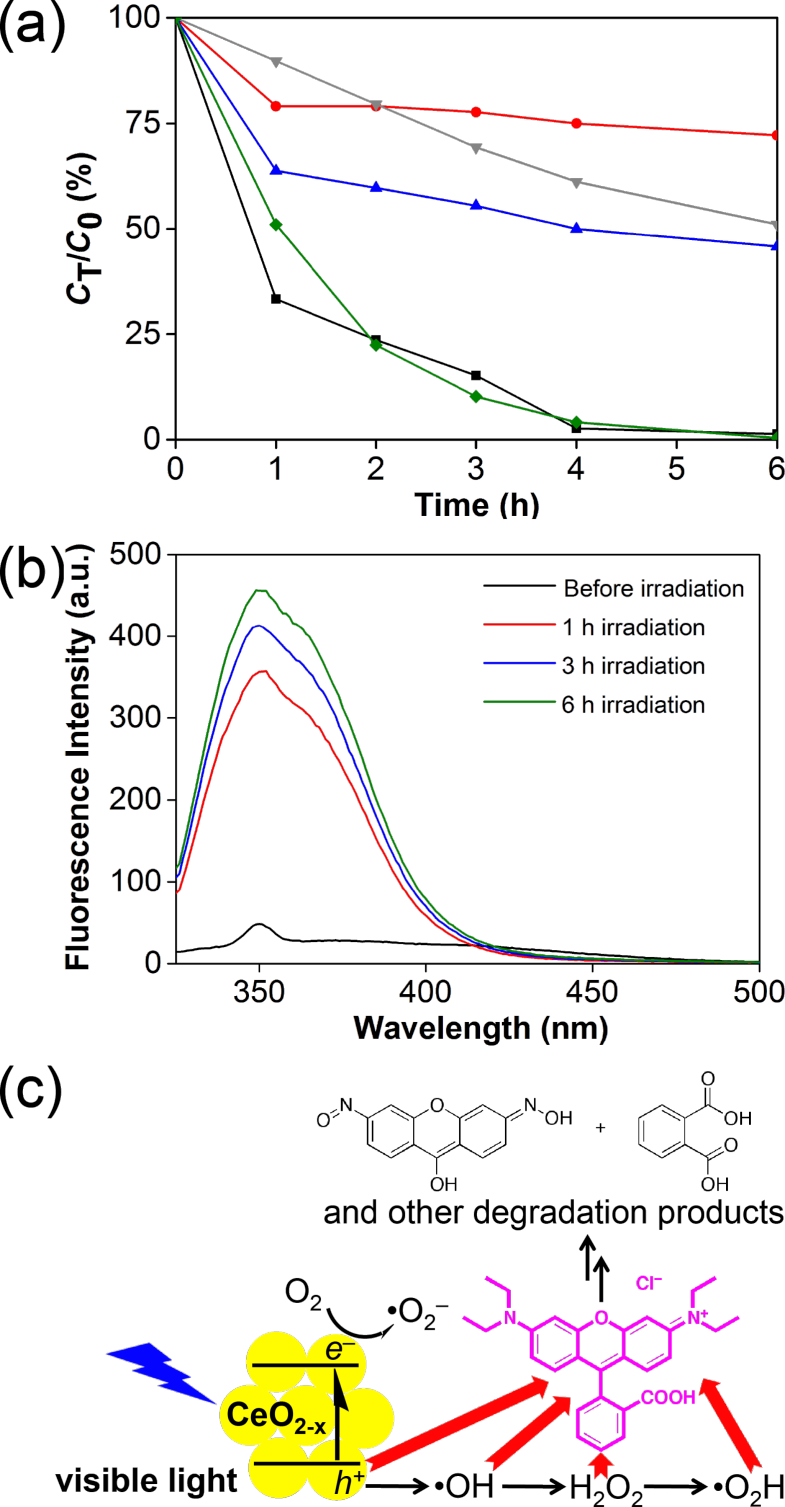
(a) Photocatalytic degradation of RhB over time at 554 nm, in the absence of scavengers (black), and the presence of *h^+^* (red), ^•^OH (blue), *e*^–^ (green), and ^•^OOH/^•^O_2_^−^ scavengers (grey). (b) Growth of the fluorescence intensity of 2-hydroxyterephthlate as a probe for hydroxyl radicals. (c) Proposed pathway for photocatalytic RhB degradation.

Chemical scavengers were employed to investigate the mechanism of the photocatalytic processes and to identify the major contributors to the photocatalytic processes. The concentration of RhB, monitored at 554 nm, was used as the proxy to identify the active agent in the decomposition of RhB. Control experiments were performed in the absence of scavengers (black line, Figure 5a). The established scavengers used include sodium oxalate for *h*^+^ (red), CrO_3_ for *e*^−^ (green), isopropanol for ^•^OH (blue), and 1,4-benzoquinone for ^•^OOH/^•^O_2_^−^ (grey, Figure 5a) [5]. The inhibition of photocatalytic activity is most pronounced in the presence of the hole scavenger, with impaired activity in the presence of both ^•^OH and ^•^OOH/^•^O_2_^−^ scavengers. Interestingly, the electron scavenger does not significantly affect the photodegradation experiments. The participation of ^•^OH radicals was confirmed with the use of sodium terephthalate as a fluorescence probe [34]. Over the course of 6 h, the fluorescence intensity due to formation of 2-hydroxyterephthalate grew [35], with a blue shift possibly due to coordination to the mesoporous cerium oxide nanoparticles (Figure 5b). These results indicate that the photocatalytic mechanism can be summarized as depicted in Figure 5c, in which *h*^+^, and downstream reactive oxygen species ^•^OH and ^•^OOH/^•^O_2_^−^, are the active agents for the chemical destruction of RhB. Superoxide radicals from the reduction of O_2_ or direct reduction by electrons from the cerium oxide appear to play secondary roles in the photocatalytic destruction of RhB.

## Conclusion

In summary, we have presented a facile, solvothermal synthesis of new mesoporous cerium oxide nanospheres, with isopropanol and ethylene glycol as the solvents and reducing agents. No expensive surfactants and templates have been used in the preparation of the earth-abundant, relatively affordable, mixed valence cerium oxide. The cerium oxide has been characterized with a suite of structural, spectroscopic, and electron microscopy techniques, confirming the high surface area, mesoporous nature, and visible-light absorption properties of the material. The visible-light photocatalytic activity in the degradation of RhB surpasses that of the commercially available CeO_2_ and P25 TiO_2_ nanopowders. With a series of radical scavengers, the mechanism of the photocatalytic activity is proposed to involve a prominent role of ^•^OH radicals as the active species in RhB degradation. This new material is a promising candidate as a robust, earth-abundant, visible-light absorbing metal oxide scaffold to be used in DSPECs and other sustainable energy applications.

## Supporting Information

The Supporting Information provides details about the synthesis of the nanospheres as well as additional experimental data.

File 1 Synthesis procedure, characterization, and dye degradation studies.

## References

[1] Andreozzi R, Caprio V, Insola A, Marotta R (1999). Catal Today.

[2] Hoffmann M R, Martin S T, Choi W, Bahnemann D W (1995). Chem Rev.

[3] Chalasani R, Vasudevan S (2013). ACS Nano.

[4] Kisch H (2013). Angew Chem, Int Ed.

[5] Wang W, Yu Y, An T, Li G, Yip H Y, Yu J C, Wong P K (2012). Environ Sci Technol.

[6] Yu K, Yang S, He H, Sun C, Gu C, Ju Y (2009). J Phys Chem A.

[7] Chen H, Nanayakkara C E, Grassian V H (2012). Chem Rev.

[8] Chen X, Mao S S (2007). Chem Rev.

[9] Hagfeldt A, Boschloo G, Sun L, Kloo L, Pettersson H (2010). Chem Rev.

[10] Hodes G, Cahen D (2012). Acc Chem Res.

[11] Kubacka A, Fernández-García M, Colón G (2012). Chem Rev.

[12] Pouretedal H R, Kadkhodaie A (2010). Chin J Catal.

[13] Vuppala V, Motappa M G, Venkata S S, Sadashivaiah P H (2012). Eur J Chem.

[14] Chueh W C, Falter C, Abbott M, Scipio D, Furler P, Haile S M, Steinfeld A (2010). Science.

[15] Chueh W C, Haile S M (2009). ChemSusChem.

[16] Scheffe J R, Steinfeld A (2012). Energy Fuels.

[17] Zhang D, Du X, Shi L, Gao R (2012). Dalton Trans.

[18] Muhich C L, Evanko B W, Weston K C, Lichty P, Liang X, Martinek J, Musgrave C B, Weimer A W (2013). Science.

[19] Ghoshal T, Fleming P G, Holmes J D, Morris M A (2012). J Mater Chem.

[20] Stetsovych V, Pagliuca F, Dvořák F, Duchoň T, Vorokhta M, Aulická M, Lachnitt J, Schernich S, Matolinová I, Veltruská K (2013). J Phys Chem Lett.

[21] Wilkens H, Schuckmann O, Oelke R, Gevers S, Schaefer A, Bäumer M, Zoellner M H, Schroeder T, Wollschläger J (2013). Appl Phys Lett.

[22] Luo H, Song W, Hoertz P G, Hanson K, Ghosh R, Rangan S, Brennaman M K, Concepcion J J, Binstead R A, Bartynski R A (2013). Chem Mater.

[23] Song W, Chen Z, Glasson C R K, Hanson K, Luo H, Norris M R, Ashford D L, Concepcion J J, Brennaman M K, Meyer T J (2012). ChemPhysChem.

[24] Alibabaei L, Luo H, House R L, Hoertz P G, Lopez R, Meyer T J (2013). J Mater Chem A.

[25] Ray S P, Cox D E (1975). J Solid State Chem.

[26] Kümmerle E A, Heger G (1999). J Solid State Chem.

[27] Ray S P, Nowick A S, Cox D E (1975). J Solid State Chem.

[28] Suresh R, Ponnuswamy V, Mariappan R (2013). Appl Surf Sci.

[29] Patterson A L (1939). Phys Rev.

[30] Kotani A, Ogasawara H (1992). J Electron Spectrosc Relat Phenom.

[31] Trudeau M L, Tschöpe A, Ying J Y (1995). Surf Interface Anal.

[32] Corma A, Atienzar P, Garcia H, Chane-Ching J-Y (2004). Nat Mater.

[33] Liang S, Wen L, Lin S, Bi J, Feng P, Fu X, Wu L (2014). Angew Chem, Int Ed.

[34] Gomes A, Fernandes E, Lima J L F C (2005). J Biochem Biophys Methods.

[35] Ishibashi K, Fujishima A, Watanabe T, Hashimoto K (2000). J Photochem Photobiol, A: Chem.

