# Supplementary material for: Mesoporous cerium oxide nanospheres for the visible-light driven photocatalytic degradation of dyes
Source: Beilstein J Nanotechnol. 2014 Apr 24;5:517–23. doi: 10.3762/bjnano.5.60 (PMC4077309; doi:10.3762/bjnano.5.60)
Supplement: File 1 — Synthesis procedure, characterization, and dye degradation studies. [file Beilstein_J_Nanotechnol-05-517-s001.pdf]

Supporting Information

for

Mesoporous cerium oxide nanospheres for the visible-light driven photocatalytic degradation of dyes

Subas K. Muduli[1,§], Songling Wang[2,§], Shi Chen[3], Chin Fan Ng[3], Cheng Hon Alfred Huan[3,4], Tze Chien Sum*[3,5,6] and Han Sen Soo*[1,6]

Address: [1]Division of Chemistry and Biological Chemistry, School of Physical and Mathematical Sciences, Nanyang Technological University, Singapore 637371, [2]Department of Chemistry, National University of Singapore, 10 Kent Ridge, Singapore 119260, [3]Division of Physics and Applied Physics, School of Physical and Mathematical Sciences, Nanyang Technological University, Singapore 637371, [4]Institute of High Performance Computing, Agency for Science, Technology and Research, 1 Fusionopolis Way, #16-16 Connexis, Singapore 138632, [5]Energy Research Institute @ NTU (ERI@N), 1 CleanTech Loop, Singapore 637141, and [6]Singapore-Berkeley Research Initiative for Sustainable Energy (SinBeRISE), 1 Create Way, Singapore 138602

Email: Tze Chien Sum* - tzechien@ntu.edu.sg, Han Sen Soo* - hansen@ntu.edu.sg

* Corresponding author

[§] These authors contributed equally to this work.

Synthesis procedure, characterization, and dye degradation studies

Experimental

Synthesis of cerium oxide nanospheres

Ceric ammonium nitrate $(NH_4)_2Ce(NO_3)_6$ (0.30 g, 0.547 mmol) was added into a solution of ethylene glycol (10 mL) and isopropanol (10 mL) under stirring. After the ceric ammonium nitrate was completely dissolved, acetic acid (1 mL) was added dropwise with vigorous stirring. In the absence of acetic acid, non-uniform aggregates of nanoparticles formed (Figure S8). After stirring for 2 h, this reaction mixture was transferred into a 100 mL Teflon-lined stainless autoclave and heated in an oven at 130 °C for 7 h, after which it was cooled to room temperature. The mesoporous cerium oxide product was collected by centrifugation, washed with ethanol and acetone, and dried at room temperature. The yield of the product was 0.087 g (~95% assuming a formula of $CeO_{1.89}$) of beige solid.

Photocatalysis

The mesoporous cerium oxide (0.030 g, 0.055 mmol) sample was added into a 10^{-5} M aqueous solution of RhB (10 mL), sonicated for 10 min, and allowed to equilibrate for 30 min. The solution was irradiated with visible light (AM 1.5 solar intensity) for photocatalytic degradation. The light source was a commercial 300 W Xenon arc lamp (ozone-free, Newport, Model no: 66984). A glass filter (Newport, Filter 20CGA-420, cut-on/cut-off wavelength tolerance: ± 5 nm, thickness: 1.1 mm) was used to cut off the UV radiation below 420 nm. During photocatalytic degradation, the distance between the light source and the surface of the rhodamin B (RhB) solution was 18 cm, the stirring rate under air was 650 rpm, and the temperature was controlled at 30 °C**.** Aliquots were removed

and centrifuged for UV–vis spectroscopy during the course of irradiation to quantify the degradation. After irradiation for 6 h, the UV–vis spectrum of the RhB remaining in solution was measured, following centrifugation to remove the mesoporous cerium oxide nanospheres. The chemical scavengers used to investigate the mechanism of the photocatalytic processes were sodium oxalate (60 mM, h^+ scavenger), isopropanol (90 mM, $^{\bullet}OH$ scavenger), 1,4-benzoquinone (1.8 mM, $^{\bullet}O_2^-$ scavenger), and CrO_3 (1.5 mM, e^- scavenger).

Characterization

The X-ray diffraction (XRD) measurements were performed on a Bruker D8 Advance X-ray diffractometer at 40 kV and 40 mA with Ni-filtered Cu Kα radiation. The morphology of the samples was determined by transmission electron microscopy (TEM) on a JEOL JEM 2010 EX instrument at an accelerating voltage of 200 keV and high-resolution transmission electron microscopy (HR-TEM) on a JEOL JEOL 2100F instrument at an accelerating voltage of 200 keV. Nitrogen adsorption–desorption isotherms were recorded using a Micromeritics ASAP 2020 physisorption analyser. UV–vis diffuse reflectance spectroscopy (DRS, $BaSO_4$ as the internal standard) was measured with a Schimadzu UV-3600 UV–vis–NIR spectrophotometer.

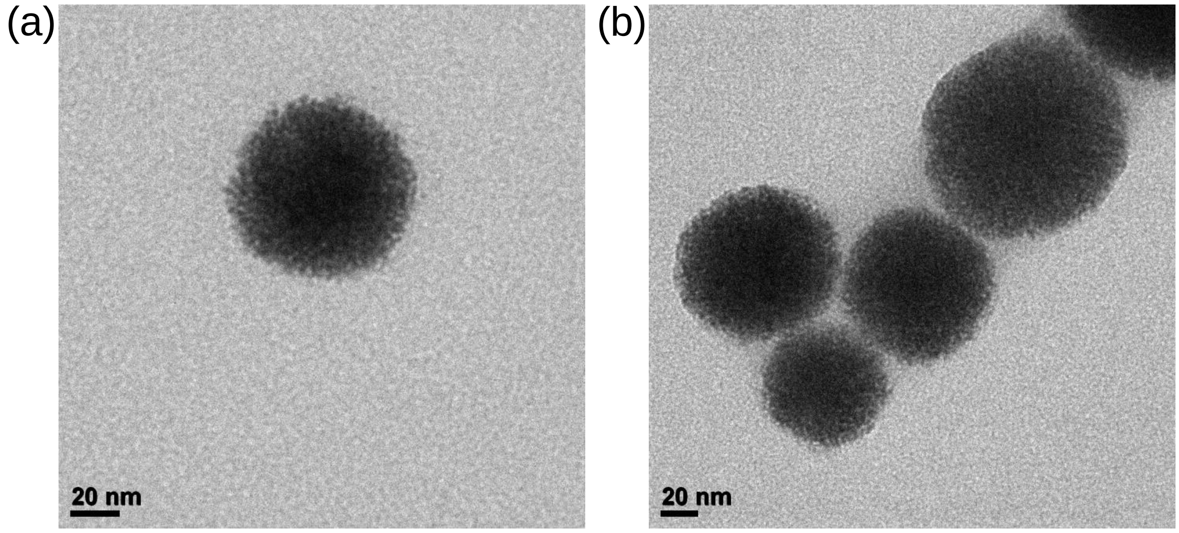

Figure S1: (a) and (b) TEM images of mesoporous cerium oxide nanospheres to show the ability to disperse readily and the consistent size distribution.

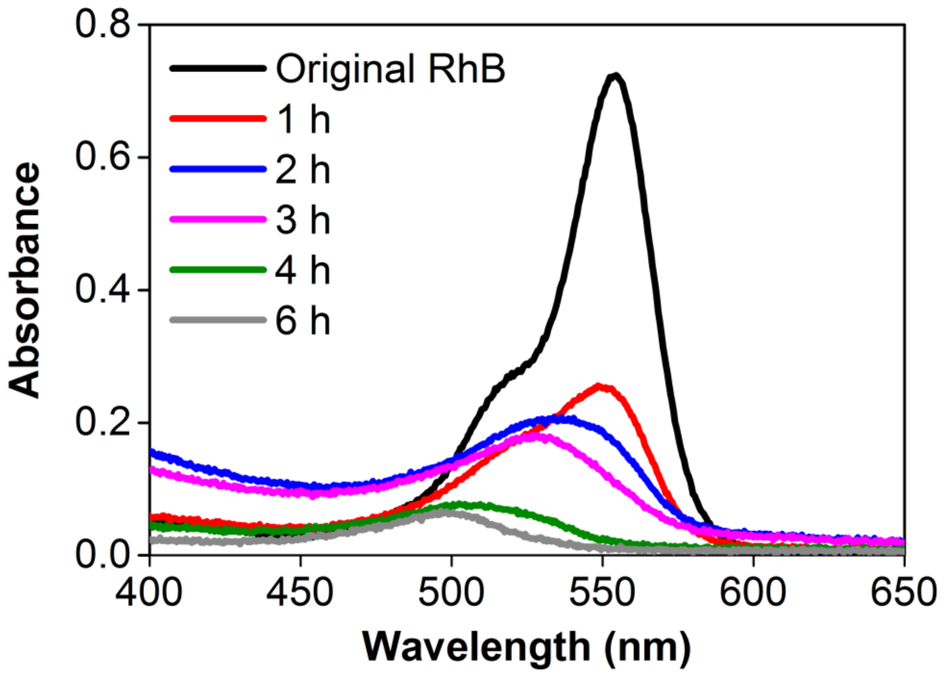

Figure S2: UV–vis absorbance spectra of the photocatalytic degradation of RhB (black, 10^{-5} M) with mesoporous cerium oxide (0.010 M) under visible light irradiation for 1 h (red), 2 h (blue), 3 h (pink), 4 h (green), and 6 h (grey).

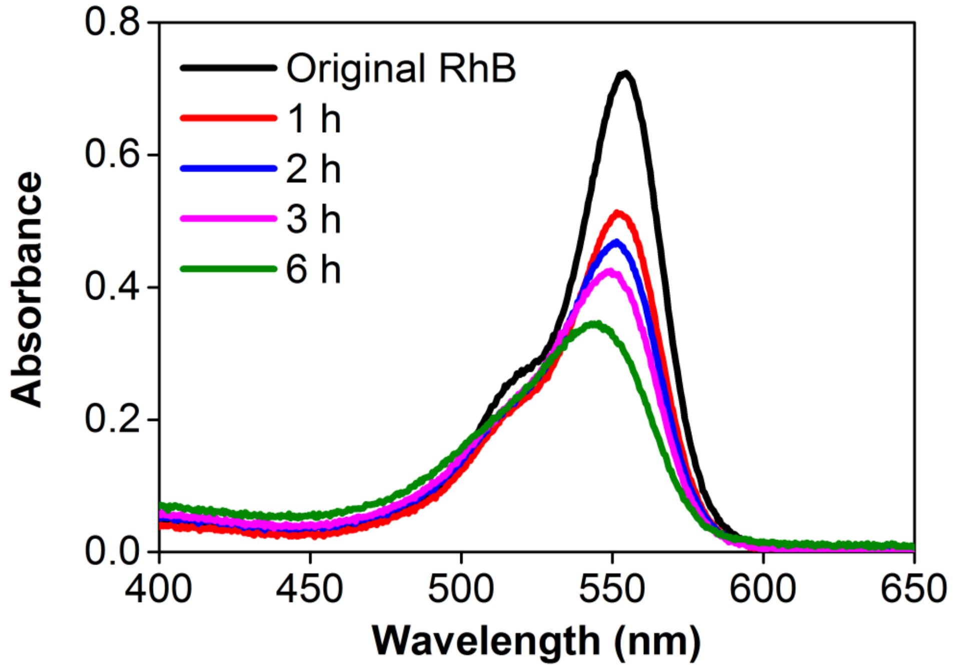

Figure S3: UV–vis absorbance spectra of the photocatalytic degradation of RhB (black, 10^{-5} M) with P25 TiO_2 (0.010 M) under visible light irradiation for 1 h (red), 2 h (blue), 3 h (pink), and 6 h (green).

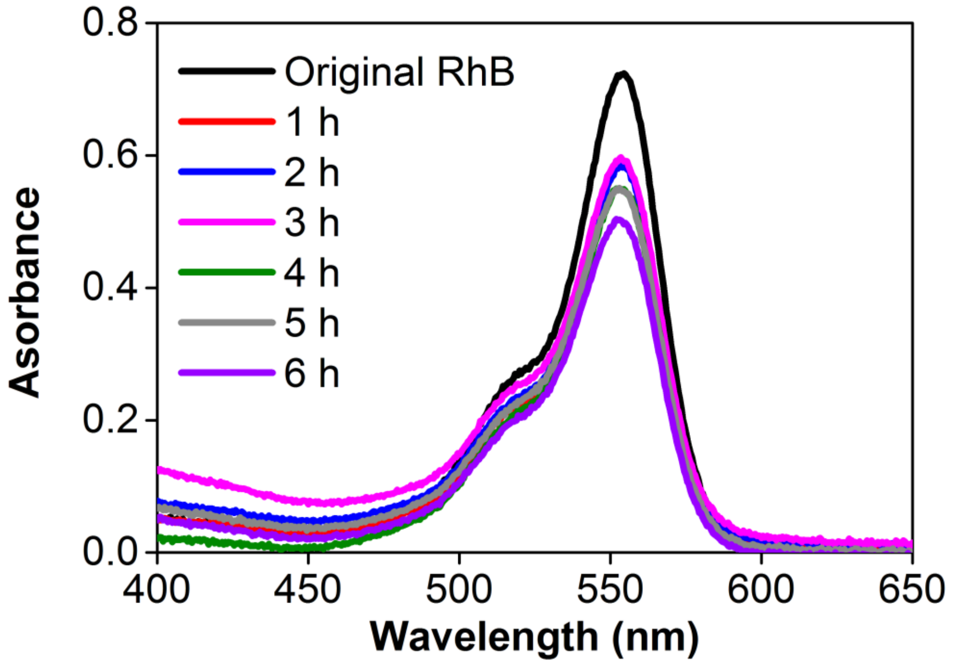

Figure S4: UV-vis absorbance spectra of the photocatalytic degradation of RhB (black, 10^{-5} M) with commercially available 7 nm CeO_2 nanopowder (0.010 M) under visible light irradiation for 1 h (red), 2 h (blue), 3 h (pink), 4 h (green), and 5 h (grey), and 6 h (violet).

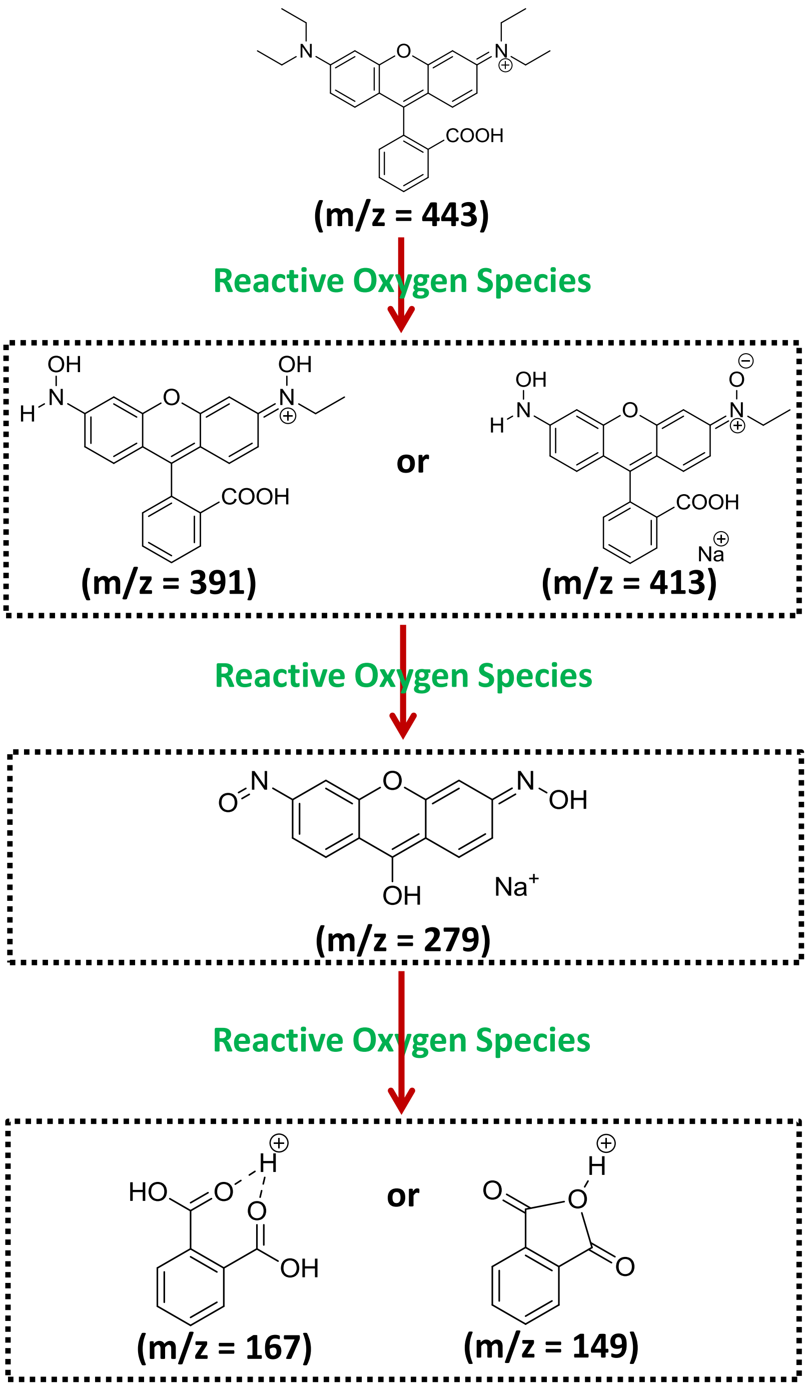

Figure S5: Schematic pathway for the photocatalytic degradation of RhB by hydroxyl radicals generated by mesoporous cerium oxide nanospheres after visible light irradiation.

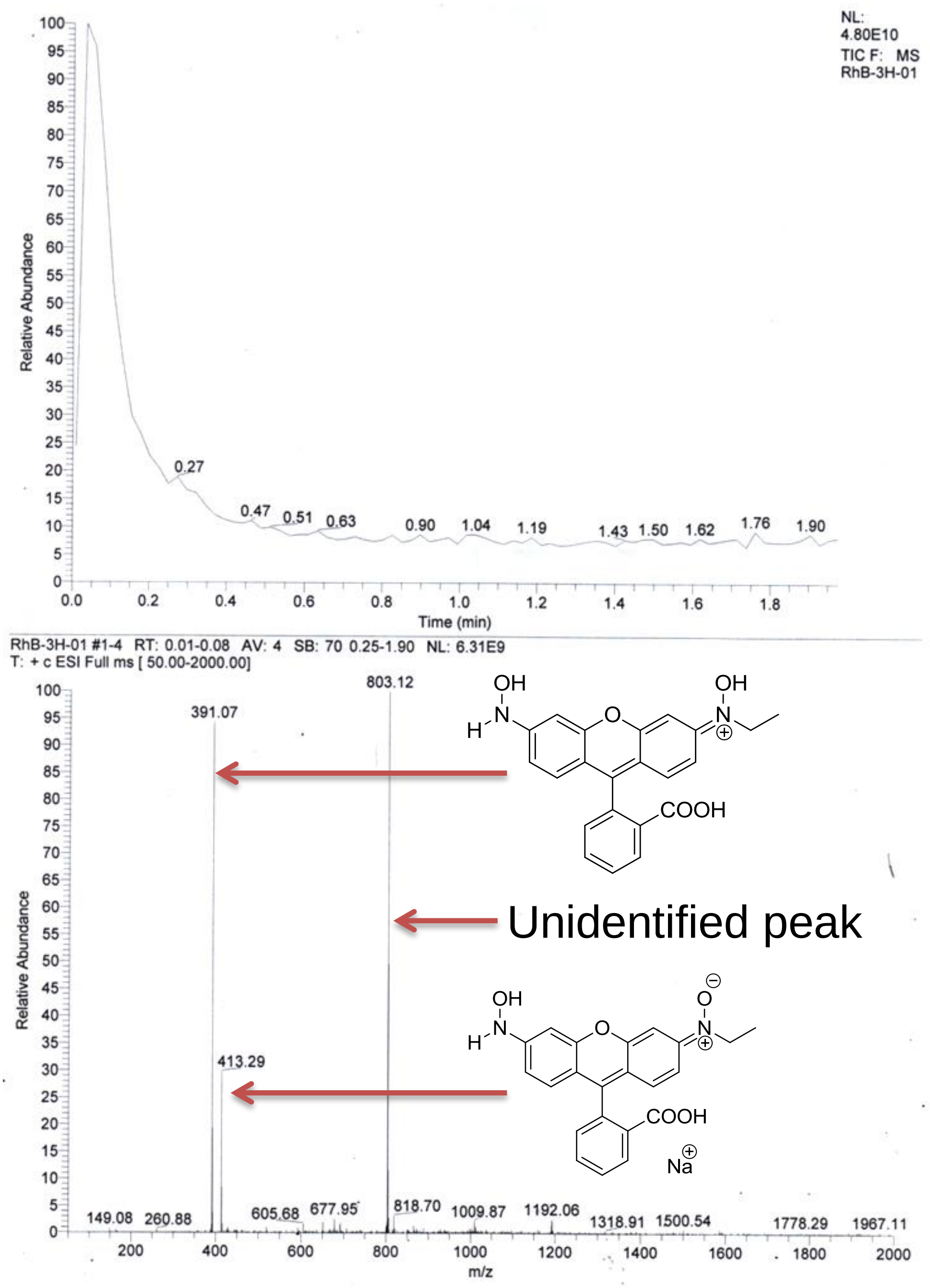

Figure S6: ESI–MS data of an aliquot from the photocatalytic degradation of RhB with mesoporous cerium oxide nanospheres after 3 h of visible light irradiation. *N*-desethyl and *N*-hydroxylated products can be observed.

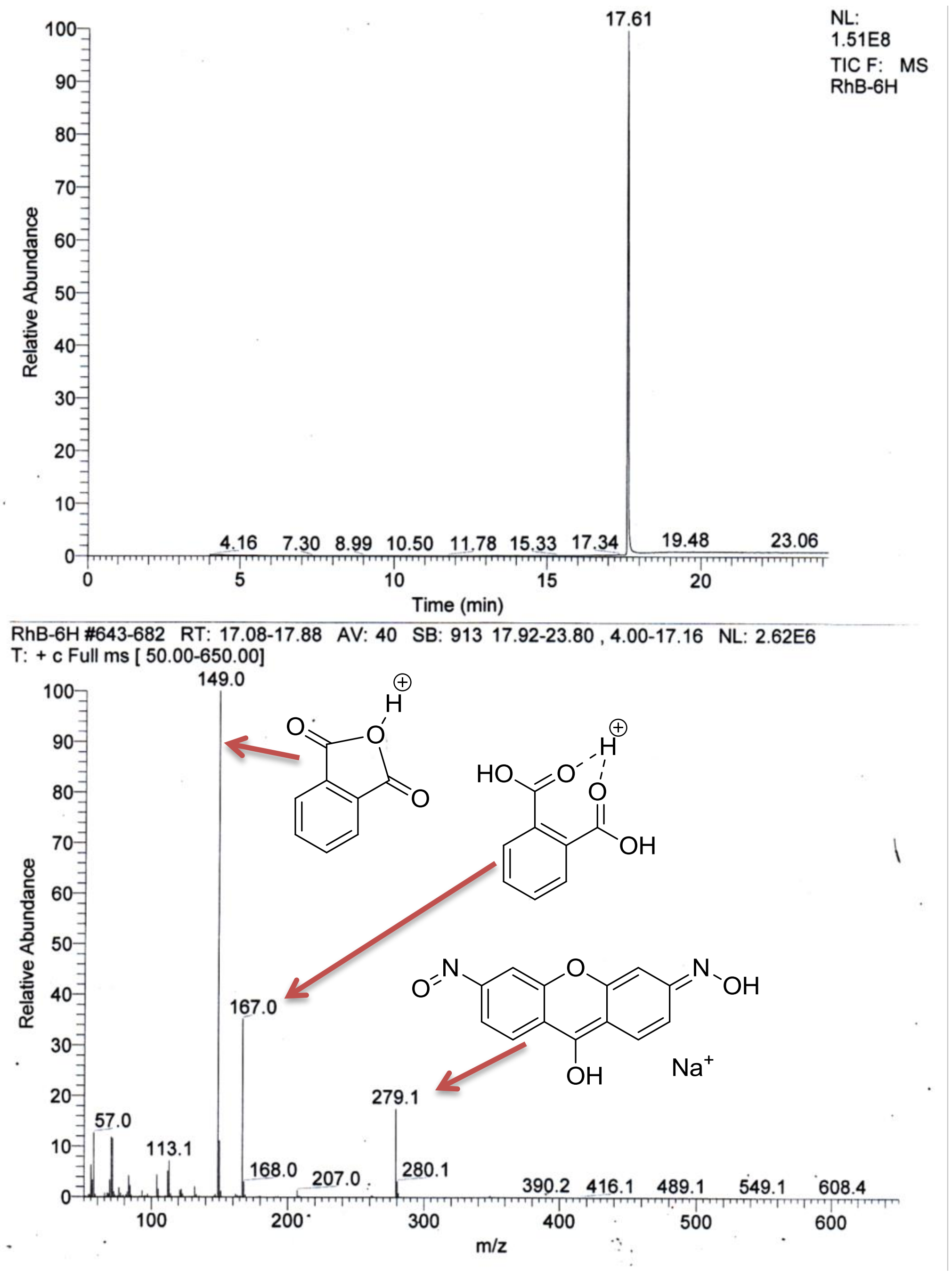

Figure S7: GC–MS data of an aliquot from the photocatalytic degradation of RhB with mesoporous cerium oxide nanospheres after 6 h of visible light irradiation. C–C cleavage products and derivatives of pthalic acid can be observed.

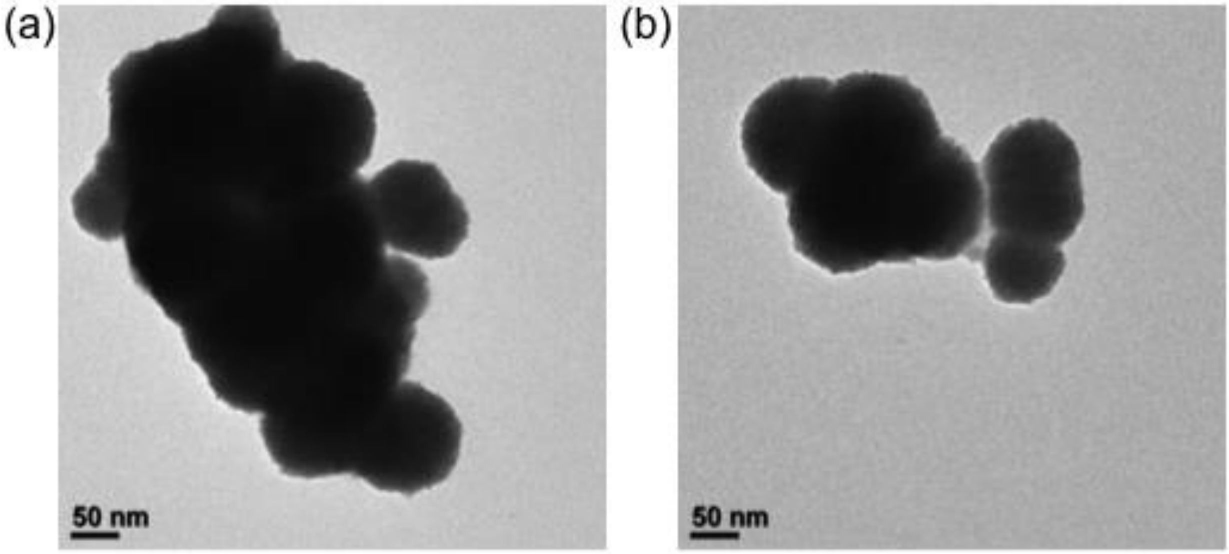

Figure S8: (a) and (b) TEM images of aggregated cerium oxide nanomaterials prepared in the absence of acetic acid that show agglomeration and irregular size distributions.
